# Antioxidant Activity and Bio-Accessibility of Polyphenols in Black Carrot (*Daucus carota* L. ssp. *sativus* var. *atrorubens* Alef.) and Two Derived Products during Simulated Gastrointestinal Digestion and Colonic Fermentation

**DOI:** 10.3390/foods10020457

**Published:** 2021-02-19

**Authors:** Gema Pereira-Caro, José Luis Ordóñez-Díaz, Elsy de Santiago, Alicia Moreno-Ortega, Salud Cáceres-Jiménez, Mónica Sánchez-Parra, Francisco Javier Roldán-Guerra, Víctor Ortiz-Somovilla, José Manuel Moreno-Rojas

**Affiliations:** 1 Department of Food Science and Health, Andalusian Institute of Agricultural and Fisheries Research and 7 Training (IFAPA), Alameda del Obispo, Avda. Menéndez-Pidal, s/n, 14004 Córdoba, Spain; mariag.pereira@juntadeandalucia.es (G.P.-C.); josel.ordonez@juntadeandalucia.es (J.L.O.-D.); elsyg.santiago@juntadeandalucia.es (E.d.S.); t22moora@uco.es (A.M.-O.); saludcaceres@gmail.com (S.C.-J.); monica.sanchez.parra@juntadeandalucia.es (M.S.-P.); franjaviroldan@gmail.com (F.J.R.-G.); victor.ortiz@juntadeandalucia.es (V.O.-S.); 2 Departamento de Bromatología y Tecnología de los Alimentos, Campus Rabanales, Ed. Darwin-anexo 9 Universidad de Córdoba, 14071 Córdoba, Spain

**Keywords:** flavonoids, anthocyanins, phenolic acids, in vitro digestion, antioxidant activity, derived products, microbial metabolites

## Abstract

Black carrot has been attracting increasing thanks to its high bioactive compound content. This study presents the polyphenol bio-accessibility of black carrot and two derived products (black carrot snack (BC snack) and black carrot seasoning (BC seasoning)) after in vitro gastrointestinal digestion and colonic fermentation. Additionally, antioxidant activity was measured by 2,2′-azinobis-(3-ethylbenzothiazoline-6-sulphonic acid) diammonium salt (ABTS), 1,1-diphenyl-2-picryl-hydrazyl (DPPH) and oxygen radical absorbance capacity (ORAC) assays. Nine flavonoids and eight anthocyanins were determined by ultra high-performance liquid chromatography high resolution mass spectrometry (UHPLC-HRMS) analysis, the predominant compounds being the hydroxycinnamic acids 3-*O*-feruloylquinic acid, 4-*O*-feruloylquinic acid and chlorogenic acid. The BC snack (108 µmol/g DW) presented the highest total polyphenol content, followed by BC seasoning (53 µmol/g DW) and black carrot (11.4 µmol/g DW). The main polyphenols still bio-accessible after in vitro digestion were the hydroxycinnamic acids, with mean recovery rates of 113 % for black carrot, 69% for BC snack and 81% for BC seasoning. The incubation of black carrot and its derived products with human faecal bacterial resulted in the complete degradation of anthocyanins and in the formation of mainly 3-(4′-hydroxyphenyl)propanoic acid as the major catabolic event. In conclusion, our results suggest that the black carrot matrix impacts significantly affects the bio-accessibility of polyphenols and, therefore, their potential health benefits.

## 1. Introduction

Black carrot (*Daucus carota* ssp. *sativus* var. *atrorubens* Alef.), also known as purple carrot, originated from countries such as Turkey, Afghanistan, Egypt, Pakistan, India and the Far East [1]. Although orange carrots are more common, the consumption of black carrots is currently increasing in Western Europe due to their benefits in human health. They present antioxidant, anti-proliferative and anti-inflammatory properties [2,3], which have been associated with their content of bioactive compounds, which are significantly related to antioxidant capacity [4,5,6]. Black carrot is characterized by an intense purple colour, which is attributed to its unique profile in specific water-soluble pigments, namely anthocyanins. The main anthocyanins in black carrots are acylated cyanidin derivatives such as cyanidin-3-feruloyl-xylosyl-glucosyl-galactoside and cyanidin-3-sinapoyl-xylosyl-glucosyl-galactoside [7,8,9,10,11,12], which have been shown to possess anticancer, antidiabetic and cardiovascular properties. Besides anthocyanins, black carrots also contain significant amounts of phenolic acids, including caffeoylquinic, chlorogenic, caffeic, sinapic, ferulic and coumaric acids [7,13,14,15].

Bioavailability is one of the factors that determine the health-promoting properties of black carrot phytochemicals. During gastrointestinal digestion, compounds may be degraded in the small intestine, or reach the colon and be metabolized by the human microbiota [16,17]. In vitro methods simulating digestion processes are widely used to study the release of compounds with the potential to be present in the intestinal brush border and be absorbed [18,19,20]. The bio-accessibility of bioactive compounds depends on their structure and binding to the food matrix.

The amount of (poly)phenolic compounds and anthocyanins in raw carrots has been reported to decrease after simulated gastrointestinal digestion [21,22]. Additionally, a decrease was found in antioxidant activity in raw black carrots after both gastric and intestinal conditions. In other studies [23,24], most of the anthocyanins in raw black carrots remained stable during gastric digestion. However, after the intestinal phase, their content drastically decreased.

In recent years, the number of new products from functional foods has increased due to their bioactive compound content. Black carrots have been used as a natural dye agent thanks to their high content of anthocyanins, which are responsible for their colour [25]. Black carrots are also processed by the food industry and consumed as soft drinks, dried snacks, juices, jellies, jams and marmalades [1]. However, during food processing, different physical and biological factors such as temperature and storage may have an effect on these bioactive compounds [26]. Kamiloglu et al. 2015 [21] reported a significant decrease in total phenolic compounds, anthocyanins and phenolic acids in jam and marmalade processed from black carrots, compared to the raw material.

Bio-accessibility studies into jam and marmalade produced from black carrots reported significant decreases in their (poly)phenol content and their antioxidant capacity after in vitro gastrointestinal digestion [27]. On the other hand, the total (poly)phenolic compound content in black carrot juice increased after simulated gastrointestinal digestion [28]. Studies into the bio-accessibility of black carrots and their derived products are very limited. Therefore, the main objective of this study was to evaluate the bio-accessibility of (poly)phenolic compounds after in vitro digestion and colonic fermentation, and to determine the antioxidant activity of black carrot and two derived products (black carrot snacks and black carrot seasoning) before and after in vitro gastrointestinal digestion.

## 2. Materials and Methods

### 2.1. Chemicals

HPLC-grade methanol (MeOH), acetone and potassium hydroxide were acquired from Panreac Applichem ITW Reagents (Darmstadt, Germany); sodium chloride and magnesium chloride hexahydrate were purchased from Fisher Scientific (Madrid, Spain); 6-hydroxy-2,5,7,8-tetramethylchroman-2-carboxylic acid (Trolox), fluorescein, 2,2′-azobis(2-amidinopropane)dihydrochloride (AAPH), 2,2′-azinobis-(3-ethylbenzothiazoline-6-sulphonic acid) diammonium salt (ABTS), sodium bicarbonate and ammonium carbonate were supplied by Sigma-Aldrich (Madrid, Spain); and potassium dihydrogen phosphate, sodium hydrogen carbonate, magnesium sulfate monohydrate and potassium hydrogen phosphate were obtained from VWR International Eurolab (Barcelona, Spain). Yeast extracts, peptone, tween 80, hemin, vitamin K, L-cysteine hydrochloride monohydrate, resazurin redox indicator and calcium chloride were acquired from Sigma-Aldrich (Madrid, Spain). α-Amylase from human saliva (300–1500 U/mg protein), pepsin (3.2–4.5 U/mg protein), pancreatin from porcine pancreas (4× UPS), bile salts and calcium chloride were purchased from Sigma-Aldrich (Madrid, Spain). Reference standard compounds including 3, 4, 5-trihydroxybenzoic acid (gallic acid), benzene-1,2-diol (catechol), 3,4-dihydroxybenzoic acid, 4-hydroxybenzoic acid, 4-hydroxy-3-methoxybenzoic acid (vanillic acid), chlorogenic acid, 3′,4′-dihydroxycinnamic acid (caffeic acid), 4′-hydroxy-3′-methoxycinnamic acid (ferulic acid), 4′-hydroxycinnamic acid (*p*-coumaric acid) and delphinidin-3-glucoside were purchased from Sigma-Aldrich (Madrid, Spain). The acetonitrile and methanol were of liquid chromatography-mass spectrometry (LC-MS) grade. The name of the phenols used in this paper has been adjusted based on the recommendations by Kay et al. [29].

### 2.2. Materials and Sample Preparation

Black carrots (*Daucus carota* L. ssp. *sativus* var. *atrorubens* Alef.) and their derived products, including the black carrot snack (BC Snack) and the black carrot seasoning (BC Seasoning), were kindly provided by the company “Esali Alimentación, S.L.” from Cuevas Bajas (Málaga, Spain). Both products derived from black carrots were obtained through a patented process involving freeze-drying techniques. This enables a porous texture to be obtained that facilitates the removal of water without the need for an increase in temperature, reducing drying time and preventing the oxidation of the product. The residual water content is less than 1%. In addition, these derived products do not contain additives or preservatives. BC seasoning is obtained by grinding the BC snack. The fresh black carrots were washed, cut into small pieces and milled using a homogenizer (SAMMIC, Madrid, Spain), before being lyophilized in a freeze dryer ECO EVO (Tred Technology S.R.L., Ripalimosani, Italy), ground and stored at −80 °C. The BC Snack was pooled and then ground and stored at −80 °C until analysis. 

### 2.3. In Vitro Gastrointestinal Digestion

An in vitro gastrointestinal digestion procedure was performed according to Moreno-Ortega et al. 2020 [30]. Three steps simulated the oral, gastric and intestinal conditions. The details of the oral, gastric and intestinal phases are described in Moreno-Ortega et al. [30]. The whole process took place in a stirred water bath (Unitronic Reciprocating Shaking Bath, model 6032011, J.P. Selecta, Barcelona, Spain) at 37 °C with 100 mL amber glass bottles containing 2 g of each sample in triplicate. During the oral phase, 2 g of lyophilized sample, 14 mL of simulated salivary fluids containing 250 µL of α-amylase (1.3 mg/mL), 0.1 mL of 0.3 M CaCl_2_ and 5.65 mL of distilled water were shaken at 37 °C for 5 min. Then, the pH was adjusted to 3 using 1 M HCl. A total of 15 mL of simulated gastric fluids was added to the samples, together with 1.19 mL of pepsin (0.1 g/mL), 0.01 mL of 0.3 M CaCl_2_ and 3.8 mL of distilled water. The mixture was incubated at 37 °C for 120 min. Finally, for the intestinal phase, 22 mL of simulated intestinal fluids were added to the samples, together with 10 mL of pancreatin solution (8 mg/mL), 5 mL of bile salts (25 mg/mL), 0.08 mL of 0.3 M CaCl_2_ and 9.92 mL of distilled water. Then, 1 M NaOH solution was used to adjust the pH to 7. The mixture was incubated for 120 min at 37 °C. Samples were taken before (BOD) and after oral digestion (AOD) and after gastric (AGD) and intestinal digestion (AID) in individual experiments. These samples were lyophilized and stored at −80 °C. The bio-accessibility index was calculated as the compound concentration after simulated gastrointestinal digestion divided by the compound concentration in non-digested samples. 

### 2.4. In Vitro Colonic Fermentation

The freeze-dried digested samples were subjected individually to in vitro fermentation to simulate the conditions in the colon following the method described by De Santiago et al. [20] and adapted to our laboratory. Details of the composition of the growth medium are described in De Santiago et al. [20]. The human faecal samples were obtained from three healthy non-smoking volunteers (three males aged between 22 and 34 and with BMIs between 20.5 and 25.4) who had not consumed antibiotics for at least 6 months prior to the study. The volunteers followed a low polyphenol diet for 48 h before the faecal sample collection. The samples were collected by the donors in plastic tubes containing an AnaeroGen sachet (Oxoid Ltd., Cambridge, UK) to maintain anaerobic conditions during transportation and were processed within 30 min of passage. Faecal slurry was prepared by homogenizing the faeces in pre-reduced phosphate buffered saline (PBS). The temperature of the incubation was set to 37 °C using a Unitronic OR circulating water-bath (JP Selecta, Abrera, Spain) and the fermentation bottles were inoculated with faecal slurry (10% *w*/*v* of fresh human faeces) for a period of 48 h. After the addition of the liophylized digested BC, BC Snack and BC Seasoning samples (2 g), the bottles were purged with oxygen free nitrogen (OFN) and sealed airtight and the anaerobiotic conditions were maintained by using a continuous OFN flow. Aliquots (1 mL) of faecal suspensions were taken after 0, 4, 8, 24 and 48 h. The samples were centrifuged at 13,500 rpm at 4 °C for 10 min and stored immediately at −80 °C until analysis. 

### 2.5. Extraction of Polyphenols from Digested Samples and from Faecal Incubates 

The extraction of polyphenols from the in vitro digested samples and the faecal samples was adapted from Ordóñez-Díaz et al. [31] with some modifications. For the in vitro digested samples, 0.25 g of lyophilized sample was homogenized with 1 mL of a methanol/acidified water mixture (80:20, *v*/*v*) with 0.1 % formic acid. The samples were centrifuged at 5000 rpm for 10 min at 4 °C, and supernatants were collected. The pellet was re-extracted with 1 mL of the same solvent as described above. All the supernatants were pooled to a final volume of 2 mL. For the fermented black carrot samples, 0.5 mL of faecal incubates were extracted using 0.5 mL of 0.1% formic acid in methanol/acidified water (80:20, *v*/*v*), vortexed and centrifuged at 5000 rpm for 10 min at 4 °C, and supernatants were collected.

### 2.6. Antioxidant Capacity Measurements

#### 2.6.1. ABST Assay

Free radical scavenging activity was measured using the ABTS discoloration method [32] with some modifications [33]. Briefly, the ABTS radical cation (ABTS^·+^) was produced by reacting ABTS with 2.45 mM of potassium persulfate solution and allowing the mixture to stand in the dark at room temperature for 12–16 h before use. The ABTS^·+^ solution was diluted with methanol to obtain a final absorbance of 0.8 ± 0.02 at 730 nm. Fresh ABTS^·+^ solution was prepared for each assay and day. Digested extracts (10 µL) were added to 190 µL of ABTS·^+^ solution in a 96-well microplate and the absorbance was measured every 20 s at 30 °C for 6 min, using a Synergy HTX Multi-Mode Microplate Reader (Biotek Instruments, Winooski, VT, USA). Methanol or Trolox were used as a blank or as standard. The percentage inhibition of absorbance versus time was plotted and the area below the curve (0–360 s) was calculated. Methanolic solutions of a known concentration of Trolox were used for calibration (0.1 to 0.8 mM). The antioxidant activity was expressed as μmol of Trolox equivalents per gram of dry sample (μmol TE/g DW). Each value is the average of three measurements.

#### 2.6.2. DPPH Assay

Free radical DPPH (1,1-diphenyl-2-picryl-hydrazyl) scavenging capacity was determined by using the methods previously described by Sánchez-Moreno et al. [34]. Briefly, digested extracts (10 µL) were added to 190 µL of DPPH 0.35 mg/mL prepared daily in a 96-well microplate. The absorbance was measured at 515 nm every minute at 30 °C for 60 min, using a Synergy HTX Multi-Mode Microplate Reader (Biotek Instruments, Winooski, VT, USA). Methanol or Trolox were used as a blank or as standard. The percentage inhibition of absorbance versus time was plotted and the area below the curve (0–60 min) was calculated. Methanolic solutions of a known concentration of Trolox were used for calibration (0.1 to 1 mM). The antioxidant activity was expressed as μmol of Trolox equivalents per gram of dry sample (μmol TE/g DW). Each value is the average of three measurements.

#### 2.6.3. Oxygen Radical Absorbance Capacity (ORAC) Assay

Oxygen radical scavenging activity was measured by the ORAC assay according to the method previously published by Huang et al. [35] and modified by Pereira-Caro et al., [33]. Briefly, 25 µL of either Trolox, digested extract or methanol as a blank were added to a 96-well microplate followed by the addition of 150 µL of fluorescein working solution (8.5 × 10^−5^ mM) prepared in 75 mM phosphate buffer (pH 7.4). The fluorescence was recorded every two minutes for 120 min at 485 and 528 nm after the addition of 30 µL of AAPH (153 mM) as peroxyl radical generator. The microplate reader (Synergy HTX Multi-Mode Microplate Reader (Biotek Instruments, Winooski, VT, USA)) was used. The final results were calculated according to Pereira-Caro et al. [33]. ORAC values are expressed as mmol Trolox equivalents per gram of dry sample (µmol TE/g DW).

### 2.7. UHPLC-HRMS Polyphenol Analysis

The polyphenols (flavonoids and anthocyanins) in the extracted samples of the in vitro digested BC, BC Snack and BC Seasoning, and in the faecal incubation samples were identified and quantified using a UHPLC-HRMS mass spectrometer system (Thermo Scientific, San José, CA, USA) consisting of a UHPLC pump, a PDA detector scanning from 200 to 600 nm, and an autosampler operating at 4 °C (ThermoFisher Scientific, San Jose, CA, USA). 

#### 2.7.1. Analysis of Flavonoids

The flavonoids were separated on a Kinetex C18 (150 × 4.6 mm i. d. 5 μm 100 A (Phenomenex, UK)) preceded by a guard pre-column of the same stationary phase and maintained at 40 °C. The mobile phases, A: acidified water 0.1% formic acid and B: acetonitrile, were pumped at a flow rate of 1.0 mL min^−1^ with a 35 min gradient starting at 3% B and maintained for 5 min, then rising to 60% B in 30 min, maintained for 3 min and then rising to 85% B in 10 min. Next, the column was equilibrated to the previous conditions within 10 min. After passing through the flow cell of the photo diode array (PDA) detector part of the column, the eluate (0.2 mL/min) entered an Exactive Orbitrap mass spectrometer (MS) (Thermo Scientific, San José, CA, USA) fitted with a heated electrospray ionization probe (HESI) operating in negative ionization mode for the determination of polyphenols. Full scans were recorded in m/z range from 100 to 1200 with a resolution of 50.000 Hz and with a full automatic gain control (AGC) target of 100,000 charges, using 2 micro-scans. Analyses were also based on scans with in-source collision-induced dissociation (CID) at 25.0 eV. The experimental conditions of the MS with HESI in negative ionization mode were a capillary temperature of 320 °C, a heater temperature of 150 °C, a sheath gas flow of 35 units, an auxiliary gas flow of 10 units, and a spray voltage of 4.0 kv. Data were acquired and processed using the Xcalibur 3.0 software.

#### 2.7.2. Analysis of Anthocyanins

The separations of anthocyanins in the digested and fermented BC, BC Snack and BC Seasoning extracts were based on the protocol previously described by Hornedo-Ortega et al. [36] with some modifications. Briefly, the separation was based on a Zorbax SB-18 column (2.1 × 100 mm d.i. 1.8 μm) (equipped with a Zorbax SB-18 1.8 μm van-guard pre-column) (Agilent, Madrid, Spain) maintained at 40 °C and eluted using two mobile phases: A: deionized water with 5% formic acid; and B: acetonitrile with 5% formic acid, over the course of 15 min at 0.3 mL min^−1^. The gradient started at 5% B, was maintained for 2 min and then rose to 100% B in 10 min. These conditions were maintained for 2 min and after 1 min returned to 5 % B, then maintained for 4 min to equilibrate the column to the initial conditions. After passing through the flow cell of the PDA detector, the column eluate went directly to an Exactive Orbitrap mass spectrometer (Thermo Scientific, San José, CA, USA) fitted with a heated electrospray ionization probe (HESI) operating in positive ionization mode for the determination of anthocyanins. Full scans were recorded in m/z range from 100 to 1000 with a resolution of 50,000 Hz and with a full AGC target of 100000 charges, using 2 micro-scans. The analyses were also based on scans with in-source collision induced dissociation (CID) at 25.0 eV. The experimental conditions of the MS with HESI in positive ionization mode were capillary temperature of 300 °C, heater temperature of 60 °C, a sheath gas flow of 20 units, an auxiliary gas flow of 2 units, and a spray voltage of 4.0 kv. Data were acquired and processed using the Xcalibur 3.0 software.

#### 2.7.3. Identification and Quantification of Flavonoids and Anthocyanins

The targeted identification of phenolic compounds and anthocyanins was achieved as follows: i) by comparing the exact mass and the retention time with available standards; and ii) in the absence of standards, compounds were tentatively identified by comparing the theoretical exact mass of the molecular ion with the measured accurate mass of the molecular ion and searched against metabolite databases including Metlin, Phenol Explorer and more general chemical databases such as PubChem and ChemSpider. Metabolites having molecular masses within the pre-specified tolerance (≤5 ppm) of the query masses were retrieved from these databases. Identifications were categorized according to the Metabolite Standards Initiative Metabolite Identification (MSIMI) levels [37]. The phenolic compounds and anthocyanins were quantified by selecting the theoretical exact mass of the molecular ion by reference to standard curves. In the absence of reference compounds, they were quantified by reference to the calibration curve of a closely related parent compound. The limits of detection (LOD) and quantification (LOQ) varied from 0.001 to 0.05 ng/µL and from 0.03 to 0.12 ng/µL, respectively. LOD and LOQ were calculated as previously reported [38].

### 2.8. Statistical Analysis

A one-way ANOVA and Tukey’s honestly significant difference (HSD) post-hoc tests were applied to identify the differences among the different phases of the in vitro gastrointestinal digestion and faecal fermentation using R software (v. 3.6.3, R Core Team, Vienna, Austria).

## 3. Results and Discussion

### 3.1. Effect of In Vitro Digestion of Black Carrot and Its Derived Products on Polyphenol Stability and Antioxidant Capacity

**Figure 1:**
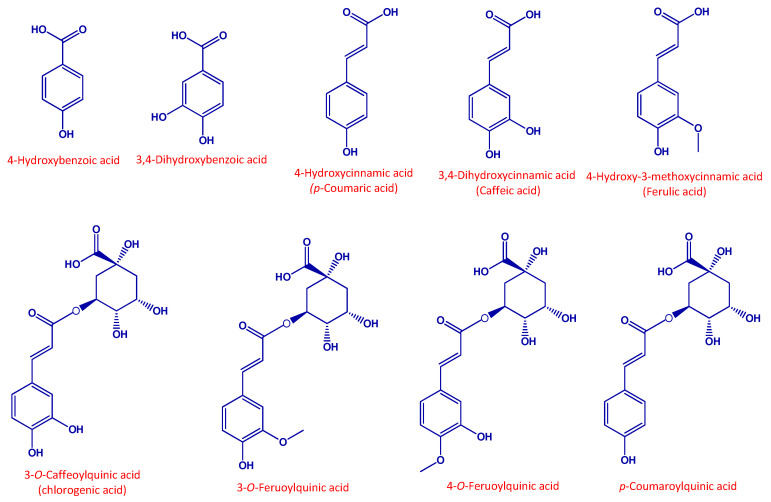
Chemical structure of the main phenolic compounds quantified in black carrot samples and derived products.

**Figure 2:**
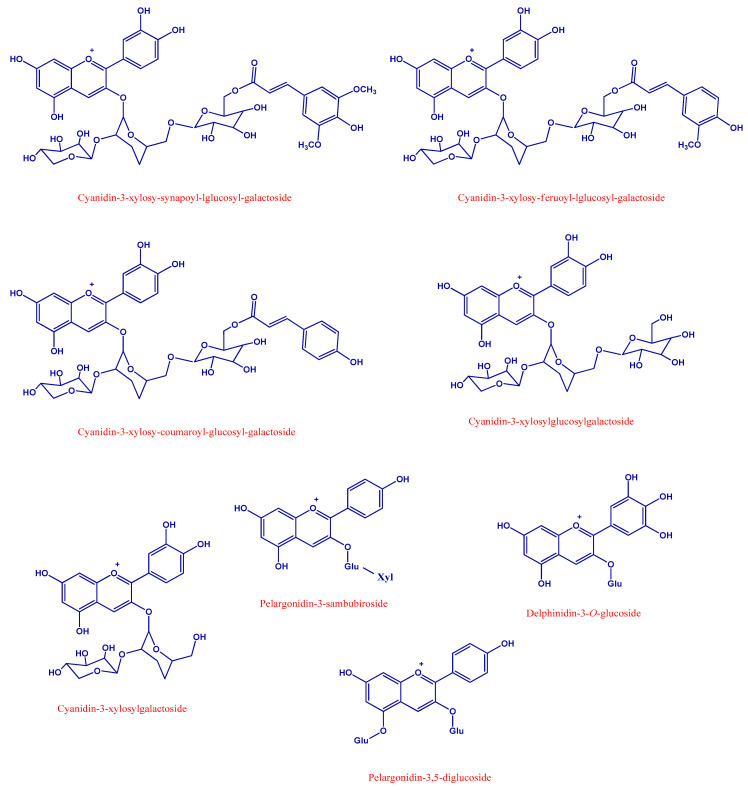
Chemical structure of the main anthocyanins quantified in black carrot samples and derived products.

Table S1 and Figures S1 and S2 (in Supporting Information) show the HPLC-HRMS characteristics and HPLC-HRMS profiles of the identified compounds. A total of 21 polyphenols were identified in black carrot and its derived products, including four hydroxybenzoic acids, seven hydroxycinnamic acids, two flavonols and eight anthocyanins. Figure 1 and Figure 2 show the chemical structures of the quantified phenolic compounds and anthocyanins in the black carrot, BC snack and BC seasoning samples, respectively. Table 1 and Table 2 provide information about the quantities of 17 polyphenols identified, including nine phenolic compounds and eight anthocyanins in black carrot and its derived products. The remaining compounds not quantified were detected in trace amounts. 

**Figure 3:**
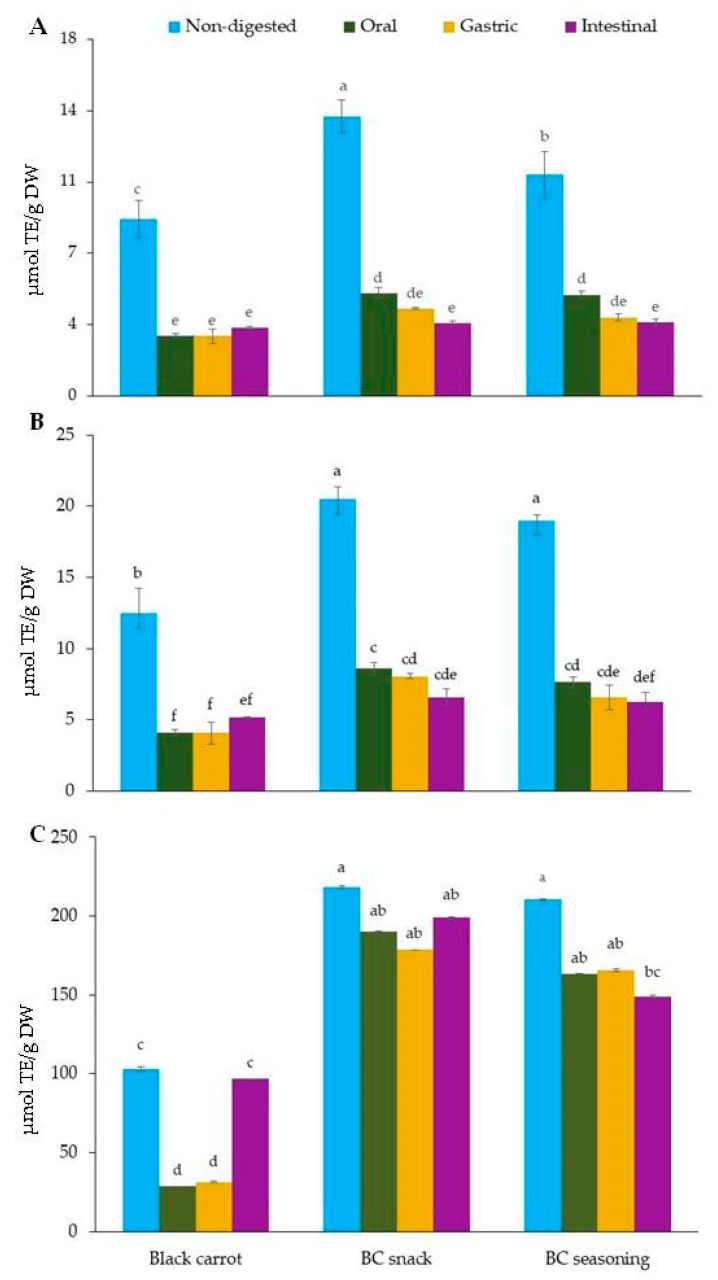
Panel (**A**–**C**) show the changes in the antioxidant activity of black carrot, black carrot snack and black carrot seasoning samples during the in vitro gastrointestinal digestion measured by the 2,2′-azinobis-(3-ethylbenzothiazoline-6-sulphonic acid) diammonium salt (ABTS), 1,1-diphenyl-2-picryl-hydrazyl (DPPH) and oxygen radical absorbance capacity (ORAC) assays, respectively. Different lowercase letters on the top of each bar denote significant differences (*p* < 0.001) within the different products (black carrot, BC snack and BC seasoning) and the digestive steps (non-digested, oral phase, gastric phase and intestinal phase) as measured by a one-way ANOVA followed the Tukey HSD post-hoc test.

Among the phenolic compounds, hydroxycinnamic acid derivatives such as choro-genic acid (2.52 µmol/g DW), 3-*O*-feruoylquinic acid (4.01 µmol/g DW) and *p*-coumaroyl-quinic acid (4.41 µmol/g DW) were the main polyphenols determined in black carrot, representing 95.8 % of the total polyphenol content, which is in accordance with previously published data [1,7]. Meanwhile, the major polyphenol in the BC snack and BC seasoning were chlorogenic acid (6.65 µmol/g DW and 3.8 µmol/g DW, respectively), 3-*O*-feruloylquinic acid (81.31 µmol/g DW and 28.04 µmol/g DW, respectively) and 4-*O*-feruloylquinic acid (11.62 µmol/g DW and 13.39 µmol/g DW, respectively), which comprised 92.3% and 86.0% of the total polyphenols in the BC snack and BC seasoning, respectively. The remaining polyphenols quantified in the black carrot and its derived products, occurring as minor compounds, were 4-hydroxybenzoic acid, 3,4-dihydroxybenzoic acid, *p*-coumaric acid, caffeic acid and ferulic acid. Overall, the total polyphenol content in the BC snack (107.88 µmol/g DW) is markedly higher than that in the BC seasoning (52.56 µmol/g DW). The black carrot presented the lowest amount of polyphenols (11.42 µmol/g DW). These results are in keeping with the antioxidant capacity determined by the ABTS, DPPH and ORAC assays (Figure 3), where the BC snack showed a significantly higher antioxidant capacity than the BC seasoning, followed by black carrot, according to the three methods used. Regarding anthocyanins, cyanidin-3-xylosyl-feruloyl-glucosyl-galactoside, followed by cyanidin-3-xylosyl-sinapoyl-glucosyl-galactoside and cyanidin-3-xylosyl-coumaroyl-glucosyl-galactoside were the main ones quantified in the black carrot, BC snack and BC seasoning, accounting for 95.2, 91.3 and 95.4 %, respectively. This anthocyanin profile, with sinapic, ferulic and coumaric acid as the predominant black carrot anthocyanins, is in line with studies in the literature, which also found acylated cyanidins [13,21]. The remaining anthocyanins consisted of five minor compounds, including cyanidin-3-xylosyl-galactoside, pelargonidin-3-sambiburoside, pelargonidin-3,5-diglucoside, cyanidin-3-xylosyl-glucosyl-galactoside and delphinidin-3-glucoside. Comparing the samples, the black carrot presented the highest total anthocyanin concentration of 194.8 nmol/g DW, while the BC snack and BC seasoning contained 8.2 and 5.2 nmol/g DW, respectively. 

The variation in the total anthocyanin and polyphenol content and in the individual anthocyanin and polyphenol profiles in black carrot and its derived products may result from the elaboration process. Indeed, several factors, such as changes in the pH, the presence of oxygen, elevated temperatures and the drying process, may affect the final concentration of polyphenols and anthocyanins in the derived products [39].

The in vitro digestion procedure indicated which compounds remain after gastrointestinal conditions and are likely to reach the colon, where they will be transformed by the resident bacteria. Table 1 and Table 2 show the respective stability of the polyphenols and anthocyanins in black carrot, BC snack and BC seasoning before (non-digested) and after oral, gastric and intestinal digestion.

After oral digestion, the concentration of phenolic compounds, including 4-hydroxybenzoic acid, 3,4-dihydroxybenzoic acid, *p*-coumaric acid, caffeic acid and ferulic acid, significantly increased in the black carrot samples, with recovery rates between 118%–435%. On the other hand, the hydroxycinnamic acids such as chlorogenic acid, 3-*O*-feruloylquinic acid, 4-*O*-feruloylquinic acid and *p*-coumaroyl-quinic acid were more unstable under the oral conditions, between 15.4%–84.6% remaining (Table 1). This can be explained by the hydrolysis of feruloyl-quinic and *p*-coumaroyl-quinic acid into ferulic acid and *p*-coumaric acid during the oral phase. The overall recovery of polyphenols after oral digestion was 6.7 µmol/g DW, representing 58.6% of the initial content. This value differs significantly from those obtained for the BC snack and BC seasoning, which showed an overall recovery of total polyphenols after oral digestion of 76.3% (82.3 µmol/g DW) and 75% (39.4 µmol/g DW), respectively. The decrease in polyphenols in both food matrices after oral digestion is mainly due to losses in specific compounds such as *p*-coumaric acid, chlorogenic acid, *O*-feruloyl-quinic acids, ferulic acid and *p*-coumaroyl-quinic acid (Table 1). Oral digestion seems to negatively affect the total concentration of polyphenols, this being more marked in the black carrot samples compared to the BC snack and BC seasoning. This behavior is also shown in the significant decrease in the antioxidant capacity after oral digestion of black carrot by ABTS, DPPH and ORAC measurements (Figure 3). In the case of the BC snack and BC seasoning, there was a significant decrease in the antioxidant activity after oral digestion, as measured by the ABTS and DPPH assays (with a 57%–66% of reduction). However, the antioxidant activity of the BC snack and BC seasoning was not affected significantly by the oral phase when this was measured by the ORAC assay (13%–19% reduction). These differences in the trend of the results could be explained by the chemistry principles upon which the antioxidant assays are based [40]. In this regard, the ORAC assay measures peroxyl-radical scavenging with a more relevant significance, while the ABTS and DPPH involve large organic radicals that present steric hindrance [41]. The gastric conditions did not greatly alter the total concentration of polyphenols with regard to the oral digestion, with recoveries of 54.8 % (6.3 µmol/g DW), 79.6% (85.8 µmol/g DW) and 73.0 % (38.4 µmol/g DW) for the black carrot, BC snack and BC seasoning, respectively. These results are in keeping with the antioxidant activity values, where there was no significant difference between the oral and gastric digestion phases by ABTS, DPPH and ORAC assays for the three food products. It is noteworthy that the concentration of hydroxycinnamate 4-*O*-feruloylquinic acid significantly increased after gastric digestion in the black carrot, with 4.1-fold increases compared to the non-digested samples, whereas this value remained stable in the BC snack and BC seasoning (Table 1). After intestinal digestion, the total polyphenol content in black carrot increased, from 6.26 µmol/g DW to 12.9 µmol/g DW, with a mean bio-accessibility of 113%. This pattern is also shown by the increase in the antioxidant capacity in the intestinal step in the black carrot samples, this increase being statistically significant by the ORAC assay. Regarding the BC snack and BC seasoning, the antioxidant capacity in the intestinal phase decreased by the ABTS and DPPH tests while the ORAC assay showed a similar antioxidant capacity for BC snack compared with the non-digested samples and a significant decrease for the BC seasoning samples. These differences can be explained by the difference in the chemistry of the three tests used, as stated above. 

The major polyphenols still bio-accessible after the digestion process were the hydroxycinnamic acids such as 3-*O*-feruloylquinic acid (4.1 µmol/g DW), 4-*O*-feruloylquinic acid (4.5 µmol/g DW) and *p*-coumaroyl-quinic acid (2.4 µmol/g DW). In the case of the BC snack and BC seasoning, the total amounts of polyphenols remaining after intestinal digestion were 74.5 µmol/g DW and 42.6 µmol/g DW, with a mean bio-accessibility of 69% and 81%, respectively. The main polyphenols were the two isomers of *O*-feruloyl-quinic acids (Table 1). This increase during the intestinal digestion phase could be associated with the release of these compounds from the plant cell walls due to the effect of the pH (pH 7) and the activity of bile salts and enzymes.

Significant changes in the anthocyanin content throughout the digestive process were found for all the samples analyzed, these being more marked in the black carrot (Table 2). After oral digestion, the total anthocyanin content in the black carrot dramatically decreased, 0.4% of the total anthocyanin content remaining, with only the cyanidin-based compounds acetylated with sinapic acid (cyanidin-3-xylosyl-sinapoyl-glucosyl-galactoside) (0.06 nmol/g DW), ferulic acid (cyanidin-3-xylosyl-feruloyl-glucosyl-galactoside) (0.62 nmol/g DW) and coumaric acid (cyanidin-3-xylosyl-coumaroyl-glucosyl-galactoside) (0.05 nmol/g DW) still present. This is in line with the premise that acetylated anthocyanins are highly stable during simulated gastrointestinal digestion, while most non-acetylated anthocyanins are degraded during this transit [42]. Due to the high impact of oral digestion on the stability of anthocyanins in black carrot, the in vitro gastric and intestinal digestion did not reveal significant changes in either in the total anthocyanin content or that of the individual anthocyanins, with a mean bio-accessibility of 0.1% and 0.5%, respectively. This result is in keeping with previous studies that reported low bio-accessibility rates of anthocyanins in mulberry (0.34%) [43], purple figs (0–5%) [44] and sour cherry (2.8%) [45]. It is also in accordance with the low serum and urine recovery of anthocyanins (< 1% dose) after the consumption of black carrot concentrate [46] and purple sweet potato [47], raspberry [48], blackcurrant [49] and blueberry [50] by humans.

These results can be explained by the fact that anthocyanins could be metabolized to non-colored forms, oxidized or even degraded into other forms which were not detected under the conditions of this study. These results contrasted notably with the effect of the digestion process on anthocyanins in the BC snack and BC seasoning products. The elaboration process needed to obtain BC snack and BC seasoning seems to have a positive effect on black carrot anthocyanin stability. For instance, the anthocyanins in the BC snack and BC seasoning were more stable under oral, gastric and intestinal digestion, with an increase in the recoveries of total anthocyanins compared to those of black carrot (Table 2). In this regard, after oral digestion, the anthocyanins remaining in the BC snack and BC seasoning accounted for 75% and 109%, respectively, cyanidin-3-xylosyl-sinapoyl-glucosyl-galactoside, cyanidin-3-xylosyl-coumaroyl-glucosyl-galactoside and cyanidin-3-xylosyl-feruloyl-glucosyl-galactoside being the main ones in both of the products derived from black carrot. These results are consistent with previously published data showing that anthocyanins in black carrot jam and marmalade are more bioavailable [21]. One hypothesis that could explain why the stability of the anthocyanidins in black carrot during the digestive process differs to that of its derived products is that transforming black carrot into powder to create the derived products results in a decreased particle size, which enlarges the surface area available for the overall extractability of some compounds from the food matrix [51].

The gastric conditions, involving pepsin activity and low pH, significantly decreased the concentration of total anthocyanins in both derived products, a total of 2.9 nmol/g DW (36 %) and 2.8 nmol/g DW (54%) of anthocyanins remaining in the BC snack and BC seasoning, respectively. On the other hand, the total anthocyanin content after intestinal digestion remained stable, with mean bio-accessibilities of 43% and 57% for the BC snack and BC seasoning, respectively (Table 2). The most bio-accessible compounds were the three acetylated cyanidins, cyanidin-3-xylosyl-feruloyl-glucosyl-galactoside achieving the highest values followed by cyanidin-3-xylosyl-sinapoyl-glucosyl-galactoside and cyanidin-3-xylosyl-coumaroyl-glucosyl-galactoside.

Similar to the results found for the polyphenol profiles, there was a significant decrease in the antioxidant activity after oral digestion compared to the non-digested samples. Figure 3 shows the effect of the in vitro digestion on the antioxidant capacity of black carrot and its derived products as measured by ABTS, DPPH and ORAC assays. After oral digestion, the antioxidant capacity did not change significantly. These results may be explained regardless of the anthocyanin content as the concentration of polyphenols is in the micromolar range while that of the anthocyanins is in the nanomolar range. 

### 3.2. Effect of Colonic Fermentation on Polyphenol Stability in Black Carrot and Its Derived Products 

**Figure 4:**
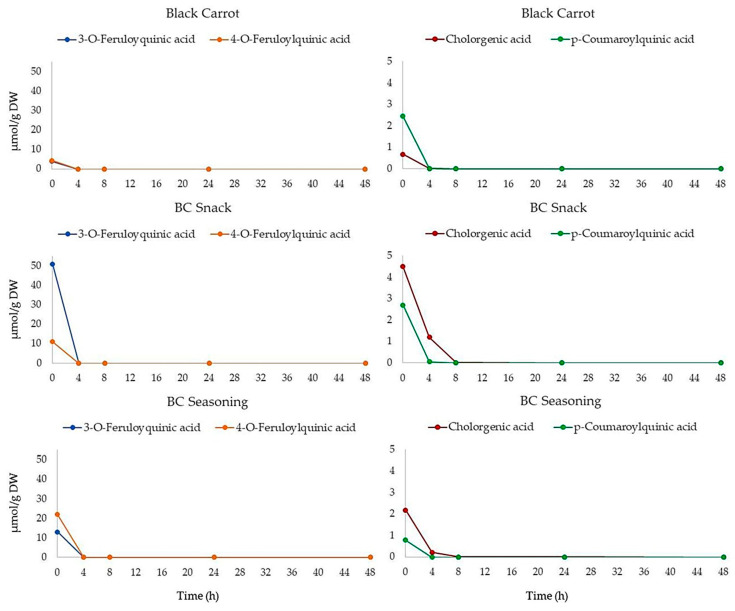
Degradation profiles of polyphenols in black carrot, BC snack and BC seasoning during 48 h of incubation with human faeces.

The digested black carrot, BC snack and BC seasoning samples were incubated with human faeces for up to 48 h and the samples were analyzed by UHPLC-HRMS. The time-course profiles of the black carrot polyphenol degradation are presented in Figure 4. The compounds present after in vitro digestion in the samples of black carrot and its derived products, including 3-*O*-feruloylquinic acid and 4-*O*-feruloylquinic acid, were degraded by the colonic bacteria within 4 h of incubation. Meanwhile, chlorogenic acid and *p*-coumaric acid were detected in low quantities after 4 h of incubation, and were completely degraded after 8 h of fermentation (Figure 4). These results are in line with those previously reported by Ludwig et al. [48], who found that chlorogenic acids in espresso coffee were totally degraded by colonic microbiota during 6 h of fermentation. 

**Figure 5:**
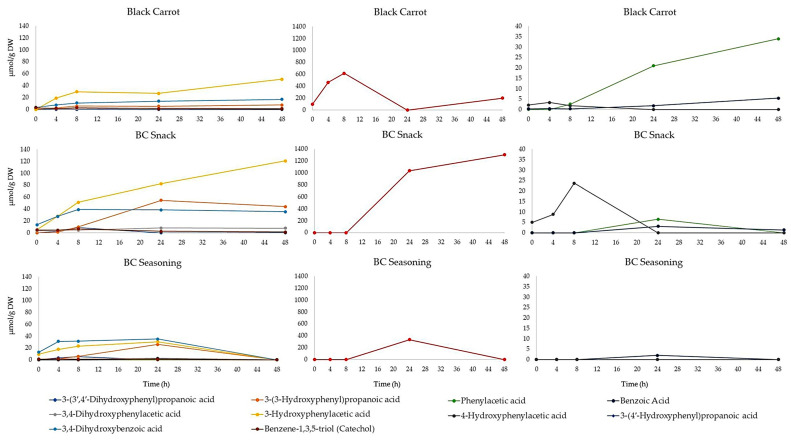
Time-course profiles of catabolites generated during faecal fermentation of black carrot, BC snack and BC seasoning during 48 h of incubation. The major polyphenols observed in the black carrot samples and its derived products before faecal fermentation were 3-*O*-feruloylquinic acid and 4-*O*-feruloylquinic acid. The first degradation event of these compounds was the catabolite 3-(4-hydroxyphenyl)propanoic acid resulting from the cleavage of the quinic acid structure, the subsequent reduction of the double bond and a de-methoxylation reaction (Figure 6). This is the main catabolite accumulated during the faecal incubation of the three black carrot products. In the black carrot incubates, its maximum concentration was reached after 8 h and declined thereafter, while the highest concentration was reached after 48 h and 24 h in the BC snack and BC seasoning, respectively. Sequentially, 3-(4′-hydroxyphenyl)propanoic acid was degraded to 4-hydroxyphenylacetic acid, via decarboxylation, and further converted to 3-phenylacetic acid, via de-hydroxylation. Small and variable levels of benzoic acid were also found, indicating that it was the product of the decarboxylation of 3-phenylacetic acid (Figure 6).

The UHPLC-HRMS analysis of the faecal samples led to the identification and quantification of ten phenolic acid catabolites. Details of the identification of these catabolites are shown in Table S2 in the Supporting Information, while their time-course profiles are shown in Figure 5. All the phenolic acids were corrected for the endogenous levels of phenolic compounds found in the faecal fermentation medium.

**Figure 6:**
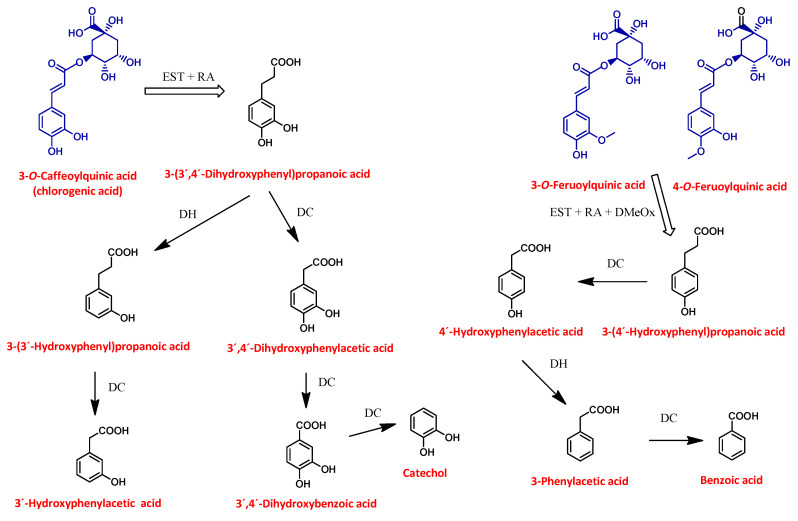
Catabolic pathway for black carrot polyphenols by the colonic microbiota. Compounds in blue presented before faecal fermentation and compounds in black were formed during faecal fermentation of black carrot. EST, esterase; RA, reductase; DH, dehydrogenase; DMeOx, de-methoxyesterase; DC, decarboxylation.

The first breakdown product of chlorogenic acid during the faecal fermentation was considered to be 3-(3′,4′-dihydrophenyl)propanoic acid, formed by the cleavage of the quinic acid structure and subsequent reduction of the double bond. Several bacteria in the colon have been shown to be able to catalyze such cleavage, including *Escherichia coli, Bifidobacterium lactis*, and *Lactobacillus gasseri* [52]. This catabolite reached its maximum concentration after 4 h for BC seasoning and after 8 h for the black carrot and BC snack, respectively (Figure 5). After 4 and 8 h of incubation, the concentration of the catabolites 3-(3-hydroxyphenyl)propanoic acid and 3,4-dihydroxyphenylacetic acid increased in the fermented medium, suggesting that they were formed from the 4′-dehydroxylation and decarboxylation of 3-(3′,4′-dihydrophenyl)propanoic acid, respectively. The catabolite 3-(3-hydroxyphenyl)propanoic was further transformed via decarboxylation to 3′-hydroxyphenylacetic acid, which appeared after 4 h of incubation, increasing throughout the faecal fermentation in the case of the BC snack, while in the black carrot and BC seasoning samples its concentration started declining after 24 h of incubation. In addition, small quantities of two other catabolites, 3,4-dihydroxybenzoic acid and catechol, were found in the incubation medium of the three black carrot products. They were formed via two consecutive decarboxylations of 3,4-dihydroxyphenylacetic acid (Figure 5). Taking all this into consideration, a catabolic pathway was proposed for the formation of phenolic catabolites from black carrot, BC snack and BC seasoning polyphenols during faecal fermentation (Figure 6). 

Overall, the total quantities of phenolic catabolites after 24 and 48 h of incubation were 81.6 ± 11 µmol/g DW and 345 ± 51 µmol/g DW for black carrot, 1236 ± 326 µmol/g DW and 1519 ± 277 µmol/g DW for BC snack, and 430 ± 77 µmol/g DW and 0 µmol/g DW for BC seasoning, respectively. The phenolic acid catabolites 3-hydroxyphenylacetic acid, phenylacetic acid and 3,4-dihydroxybenzoic acid, comprising 87% of the total catabolites, were the major end products after 24 h of fermentation. The same catabolites, together with 3-(4′-hydroxyphenyl)propanoic acid, represented 90% of the total catabolites after 48 h of incubation. In contrast, the major end product of the BC snack and BC seasoning was the catabolite 3-(4′-hydroxyphenyl)propanoic acid, which comprised 84 and 86% after 24 h and the 77% of the total catabolites after 48 h, respectively.

## 4. Conclusions

This study reports the stability and bio-accessibility of the phytochemicals in black carrot and two derived products, BC snack and BC seasoning, during in vitro digestion and faecal fermentation with human microbiota. Black carrot anthocyanins were mostly affected by the oral phase of the gastrointestinal digestion, these being more bio-accessible in the BC snack and BC seasoning. Meanwhile, hydroxycinnamic acids, which are present in significantly higher quantities compared to anthocyanins, are more stable under the digestion process. After faecal fermentation, the parent polyphenols in the black carrot samples and its derived products were completely degraded after 4 h of incubation, being converted to 10 phenolic acid catabolites, highlighting their extensive transformation by the human faecal microbiota. The different catabolic profiles among the black carrot and the BC snack and BC seasoning suggested that the stability and bio-accessibility of polyphenols depends largely on the food matrix. Nevertheless, the catabolite 3-(4′-hydroxyphenyl)propanoic acid, which is the major compound present during faecal fermentation, merits further investigation. It is potentially responsible for the health benefits at colonic level associated with the consumption of black carrot and its derived products. 

## Figures and Tables

**Table 1:** Quantities (µmol/g DW) of polyphenols determined in black carrot, black carrot snack and black carrot seasoning before (non-digested) and after oral, gastric and intestinal in vitro digestion. Different letters within a column denote a significant difference (*p* < 0.05) as measured by one-way ANOVA followed by the Tukey honestly significant difference (HSD) post-hoc test. n.d.: not detected.

	4-Hydroxybenzoic Acid	3,4-Dihydroxybenzoic Acid	*p*-Coumaric Acid	Caffeic Acid	Chlorogenic Acid	3-O-Feruloylquinic Acid	4-O-Feruloylquinic Acid	Ferulic Acid	*p*-Coumaroyl Quinic Acid	Total
Black Carrot										
Non-digested	0.13 ± 0.02 ^c^	0.22 ± 0.04 ^d^	0.01 ± 0.01 ^b^	0.02 ± 0.01 ^c^	2.52 ± 0.08 ^a^	4.01 ± 0.02 ^a^	0.06 ± 0.01 ^c^	0.04 ± 0.01 ^b^	4,41 ± 0.8 ^a^	11.4 ± 0.3 ^a^
Oral Digestion	0.37 ± 0.01 ^a^	0.44 ± 0.05 ^c^	0.03 ± 0.01 ^a^	0.09 ± 0.01 ^a^	0.39 ± 0.03 ^c^	3.4 ± 0.7 ^b^	n.d.	0.05 ± 0.01 ^a^	1.93 ± 0.02 ^c^	6.7 ± 0.8 ^b^
%	297	199	342	435	15.4	85	0	118	44	58.6
Gastric Digestion	0.27 ± 0.01 ^b^	0.65 ± 0.01 ^b^	0.02 ± 0.01 ^b^	0.03 ± 0.01 ^b^	0.55 ± 0.06 ^b^	2.5 ± 0.4 ^c^	0.25 ± 0.06 ^b^	0.03 ± 0.01 ^c^	1.9 ± 0.7 ^c^	6.3 ± 0.6 ^b^
%	212	297	190	135	21.8	63	405	65	44	54.8
Intestinal Digestion	0.33 ± 0.01 ^a^	0.73 ± 0.02 ^a^	0.02 ± 0.00 ^b^	0.04 ± 0.00 ^b^	0.68 ± 0.03 ^b^	4.08 ± 0.04 ^a^	4.5 ± 0.2 ^a^	0.05 ± 0.01 ^a^	2.44 ± 0.03 ^b^	12.9 ± 0.3 ^a^
%	259	333	285	218	26.9	102	7231	136	55	113
Black Carrot Snack										
Non-digested	0.87 ± 0.05 ^c^	3.2 ± 0.1 ^a^	0.03 ± 0.01 ^a^	0.27 ± 0.04 ^a^	6.6 ± 0.2 ^a^	81.3 ± 0.7 ^a^	11.6 ± 0.8 ^b^	0.56 ± 0.04 ^a^	3.34 ± 0.03 ^a^	108 ± 2 ^a^
Oral Digestion	1.30 ± 0.09 ^b^	3.0 ± 0.2 ^a^	0.02 ± 0.00 ^a^	0.25 ± 0.02 ^b^	5.3 ± 0.4 ^b^	60 ± 11 ^b^	9 ± 1 ^c^	0.43 ± 0.09 ^c^	2.6 ± 0.6 ^b^	82 ± 13 ^b^
%	149	92	74	90	80	74	79	77	79	76
Gastric Digestion	2.0 ± 0.1 ^a^	2.8 ± 0.0 ^b^	n.d.	0.2 ± 0.0 ^c^	4.6 ± 0.1 ^c^	60 ± 4 ^b^	13 ± 1 ^a^	0.5 ± 0.1 ^b^	3.1 ± 0.1 ^a^	85 ± 5 ^b^
%	230	87.6	0	91	69	74	109	81	93	80
Intestinal Digestion	1.8 ± 0.0 ^a^	2.8 ± 0.1 ^b^	n.d.	0.2 ± 0.0 ^c^	4.5 ± 0.1 ^c^	51 ± 2 ^c^	11.1 ± 0.5 ^b^	0.5 ± 0.0 ^b^	2.7 ± 0.8 ^b^	74 ± 3 ^c^
%	206	86	0	83	68	62	95	92	82	69
Black Carrot Seasoning										
Non-digested	2.14 ± 0.05 ^a^	2.8 ± 0.2 ^a^	0.01 ± 0.00	0.05 ± 0.01	3.80 ± 0.08 ^a^	28 ± 4 ^a^	13.4 ± 0.8 ^b^	0.34 ± 0.07 ^a^	1.98 ± 0.05 ^a^	53 ±2 ^a^
Oral Digestion	1.8 ± 0.1 ^b^	2.4 ± 0.1 ^b^	0.01 ± 0.00	0.05 ± 0.01	3.1 ± 0.8 ^b^	21 ± 7 ^b^	9.0 ± 0.5 ^c^	0.22 ± 0.01 ^b^	1.4 ± 0.5 ^b^	39 ± 9 ^b^
%	82	86	100	100	83	76	67	66	72	75
Gastric Digestion	1.9 ± 0.6 ^b^	2.7 ± 0.4 ^a b^	n.d.	n.d.	2.3 ± 0.4 ^c^	17 ± 6 ^c^	13 ± 1 ^b^	0.2 ± 0.0 ^b^	1.1 ± 0.4 ^c^	38 ± 9 ^b^
%	88.6	97	0	0	60	62	94	58	56	73
Intestinal Digestion	1.9 ± 0.3 ^b^	2.4 ± 0.5 ^b^	0.01 ± 0.00	0.05 ± 0.01	2.2 ± 0.1 ^c^	13 ± 1 ^d^	22 ± 8 ^a^	0.11 ± 0.01 ^c^	0.79 ± 0.05 ^d^	43 ±10 ^ab^
%	89	87	100	100	58	47	164	32	40	81

**Table 2:** Quantities (nmol/g DW) of anthocyanins determined in black carrot, black carrot snack and black carrot seasoning before (non-digested) and after oral, gastric and intestinal in vitro digestion. Xyl: xylosyl; Glu: glucoside; Gal: galactoside.

	Cyanidin-3-xyl-gal	Cyanidin-3-xyl-coumaroyl-glu-gal	Cyanidin-3-xyl-feruloyl-glu-gal	Cyanidin-3-xyl-sinapoyl-glu-gal	Pelargonidin-3-sambiburoside	Pelargonidin-3,5-diglu	Cyanidin-3-xyl-glu-gal	Delphinidin-3-glu	Total
Black Carrot									
Non-digested	7 ± 1	15 ± 1 ^a^	134 ± 11 ^a^	35.7 ± 0.9 ^a^	0.15 ± 0.01	0.14 ± 0.01	1.8 ± 0.5	0.05 ± 0.01	195 ± 15 ^a^
Oral Digestion	n.d.	0.05 ± 0.01 ^b^	0.62 ± 0.05 ^b^	0.06 ± 0.01 ^b^	n.d.	n.d.	n.d.	n.d.	0.7 ± 0.1 ^b^
%	0	0.3	0.5	0.2	0	0	0	0	0.4
Gastric Digestion	n.d.	0.009 ± 0.001 ^c^	0.15 ± 0.02 ^c^	0.009 ± 0.001 ^c^	n.d.	n.d.	n.d.	n.d.	0.2 ± 0.0 ^c^
%	0	0.1	0.1	0.05	0	0	0	0	0.1
Intestinal Digestion	n.d.	0.07 ± 0.02 ^b^	0.9 ± 0.1 ^b^	0.05 ± 0.02 ^b^	n.d.	n.d.	n.d.	n.d.	1.1 ± 0.2 ^b^
%	0	0.4	0.7	0.1	0	0	0	0	0.5
Black Carrot Snack									
Non-digested	0.28 ± 0.02 ^a^	0.78 ± 0.07 ^a^	4.76 ± 0.01 ^a^	1.94 ± 0.01 ^a^	0.009 ± 0.001 ^b^	0.023 ± 0.001 ^b^	0.376 ± 0.002 ^a^	0.02 ± 0.01 ^a^	8.2 ± 0.1 ^a^
Oral Digestion	0.16 ± 0.01 ^b^	0.54 ± 0.03 ^b^	3.6 ± 0.2 ^b^	1.5 ± 0.1 ^b^	0.012 ± 0.001 ^a^	0.01 ±0.01 ^a^	0.21 ± 0.02 ^b^	0.01 ± 0.01 ^b^	6.1 ± 0.5 ^b^
%	59	70	77	78	133	61	57	46	75
Gastric Digestion	0.07 ± 0.02 ^c^	0.24 ± 0.02 ^c^	1.7 ± 0.2 ^c^	0.71 ± 0.02 ^c^	n.d.	n.d.	0.17 ± 0.04 ^c^	n.d.	2.9 ± 0.3 ^c^
%	27	31	36	37	0	0	45	0	36
Intestinal Digestion	0.036 ± 0.001 ^d^	0.27 ± 0.04 ^c^	2.2 ± 0.2 ^c^	0.83 ± 0.05 ^c^	n.d.	n.d.	0.15 ± 0.02 ^c^	n.d.	3.5 ± 0.3 ^c^
%	13	35	47	43	0	0	41	0	43
Black Carrot Seasoning									
Non-digested	0.009 ± 0.010 ^b^	0.30 ± 0.02 ^a^	3.0 ± 0.5 ^b^	1.6 ± 0.4 ^a^	n.d.	n.d.	0.06 ± 0.02	0.09 ± 0.01	5 ± 1 ^a^
Oral Digestion	0.03 ± 0.02 ^a^	0.26 ± 0.01 ^b^	3.7 ± 0.3 ^a^	1.6 ± 0.2 ^a^	n.d.	n.d.	n.d.	n.d.	5.7 ± 0.5 ^a^
%	34	88	124	101	0	0	0	0	109
Gastric Digestion	0.02 ± 0.01 ^a^	0.14 ± 0.01 ^c^	1.86 ± 0.01 ^c^	0.80 ± 0.08 ^b^	n.d.	n.d.	n.d.	n.d.	2.8 ± 0.1 ^b^
%	21	48	62	48	0	0	0	0	54
Intestinal Digestion	0.009 ± 0.001 ^b^	0.14 ± 0.01 ^c^	1.9 ± 0.1 ^c^	0.84 ± 0.02 ^b^	n.d.	n.d.	n.d.	n.d.	2.9 ± 0.1 ^b^
%	10	46	65	51	0	0	0	0	57

Different letters within a column denote a significant difference (*p* < 0.05) as measured by one-way ANOVA followed by the Tukey HSD post-hoc test. n.d.: not detected.

## Supplementary Materials

The following are available online at https://www.mdpi.com/2304-8158/10/2/457/s1, Table S1: UHPLC-HRMS characteristics of the polyphenols identified in black carrot and its derived products (black carrot snack and black carrot seasoning). Table S2. UHPLC-HRMS characteristics of phenolic acid catabolites identified in black carrot and its derived products in faecal incubates. Figure S1: Representative UHPLC-HRMS profile of polyphenols in black carrot. For peak identification see Table S1. Figure S2: Representative UHPLC-HRMS profile of anthocyanins in black carrot. For peak identification see Table S1.
