# Supplementary material for: Antioxidant Activity and Bio-Accessibility of Polyphenols in Black Carrot (Daucus carota L. ssp. sativus var. atrorubens Alef.) and Two Derived Products during Simulated Gastrointestinal Digestion and Colonic Fermentation
Source: Foods. 2021 Feb 19;10(2):457. doi: 10.3390/foods10020457 (PMC7922073; doi:10.3390/foods10020457)
Supplement: Supplementary file 1 [file foods-10-00457-s001.pdf]

Table S1. UHPLC-HRMS Characteristics of the Polyphenols Identified in Black Carrot and its by-products (black carrot snack and black carrot seasoning).

Peak	Compounds	Chemical Formula	[m/z]⁻ Theoretical	Error (ppm)	RT (min)	MSIMI level[b]
	Hydroxybenzoic acids					
1	Gallic acid	$C_7H_6O_5$	169.0131	1.12	3.2	1
2	3.4-Dihydroxybenzoic acid	C7H6O4	153.0182	1.6	5.9	1
3	4-Hydroxybenzoic acid	C7H6O3	137.0243	1.82	9.3	1
4	Vanillic acid		167.0338		11.23	1
	Hidroxycinnamic acids					
5	Chlorogenic acid	C16H18O9	353.0867	1.63	11.7	1
6	Caffeic acid	C9H8O4	179.0351	2.31	11.8	1
7	*p*-Coumaric acid	C9H8O3	163.0395	2.45	13.9	1
8	Ferulic acid	C10H10O4	193.0516	3.29	11.7	1
9	*3-O*-Feruoylquinic acid	C17H20O9	367.1024	1.31	14.4	2
10	*4-O*-Feruoylquinic acid	C17H20O9	367.1024	1.31	14.8	2
11	*p*-Coumaroyl quinic acid	C16H18O8	337.0917	1.95	13.21	2
	Flavonols					
12	Quercetin-3-galactoside	C21H20O12	463.0871	3.06	15.9	2
13	Quercetin-3-diglucoside	C27H30O17	625.1412	2.16	14.52	2
	Anthocyanins					
14	Cyanidin-3-xylosyl-glucosyl-galactoside	C32H38O20	743.2039[a]	-2.12	5.1	2
15	Cyanidin-3-xylosyl-galactoside	C26H28O15	581.1497[a]	-0.6	5.3	2
16	Cyanidin-3-xylosyl(sinapoylglucosyl)galactoside	C43H48O23	949.2608[a]	-1.26	5.6	2
17	Cyanidin-3-xylosyl(feruoylglucosyl)galactoside	C42H46O23	919.2503[a]	-2.75	5.7	2
18	Cyanidin-3-xylosyl(coumaroylglucosyl)galactoside	C41H44O22	889.2397[a]	-0.76	5.7	2
19	Pelargonidin-3.5-diglucoside	C27H30O15	595.1641[a]	-0.53	5.7	2
20	Pelargonidin-3-sambiburoside	C26H28O14	565.1544[a]	-1.42	5.6	2
21	Delphinidin-3-glucoside	C21H20O12	465.1028[a]	-0.87	6.4	1

[a] $[m\text{-}z]^+$ for anthocyanins. [b] Annotation from the Summer et al. Compounds identified at MSIMI level 1 means there was a commercially available standard. Compounds identified at MSIMSI level 2 means it was tentativelly identified. RT: retention time.

Table S2. UHPLC-HRMS Characteristics of Phenolic Acid Catabolites Identified in Black Carrot and its by-products Faecal Incubates

Rt (min)	Catabolites	Chemical Formula	$[m/z]^-$ Theoretical	Error (ppm)	MSIMI level[a]
10.1	3-(3′,4′-Dihydroxyphenyl)propanoic acid	$C_9H_{10}O_4$	181.0495	0.14	1
13.6	3-(3′-Hydroxyphenyl)propanoic acid	$C_9H_9O_3$	165.0546	0.01	1
12.9	3-(4′-Hydroxyphenyl)propanoic acid	$C_9H_9O_3$	165.0546	-0.01	1
13.3	3,4-Dihydroxyphenylacetic acid	$C_8H_8O_4$	167.0338	0.005	1
10.5	3-Hydroxyphenylacetic acid	$C_8H_8O_3$	151.0389	-0.09	1
11.5	4-Hydroxyphenylacetic acid	$C_8H_8O_3$	151.0389	-0.01	1
15.1	Phenylacetic acid	$C_8H_8O_2$	135.0400	1.25	1
5.7	3,4-Dihydroxybenzoic acid	$C_7H_6O_4$	153.0182	-0.01	1
15.2	Benzoic acid	$C_7H_6O_2$	121.0284	1.02	1
2.3	Benzene-1,2-diol (catecol)	$C_6H_6O_2$	109.0284	2.01	1

[a] Annotation from the Summer et al. Compounds identified at MSIMI level 1 means there was a commercially available standard. RT: retention time.

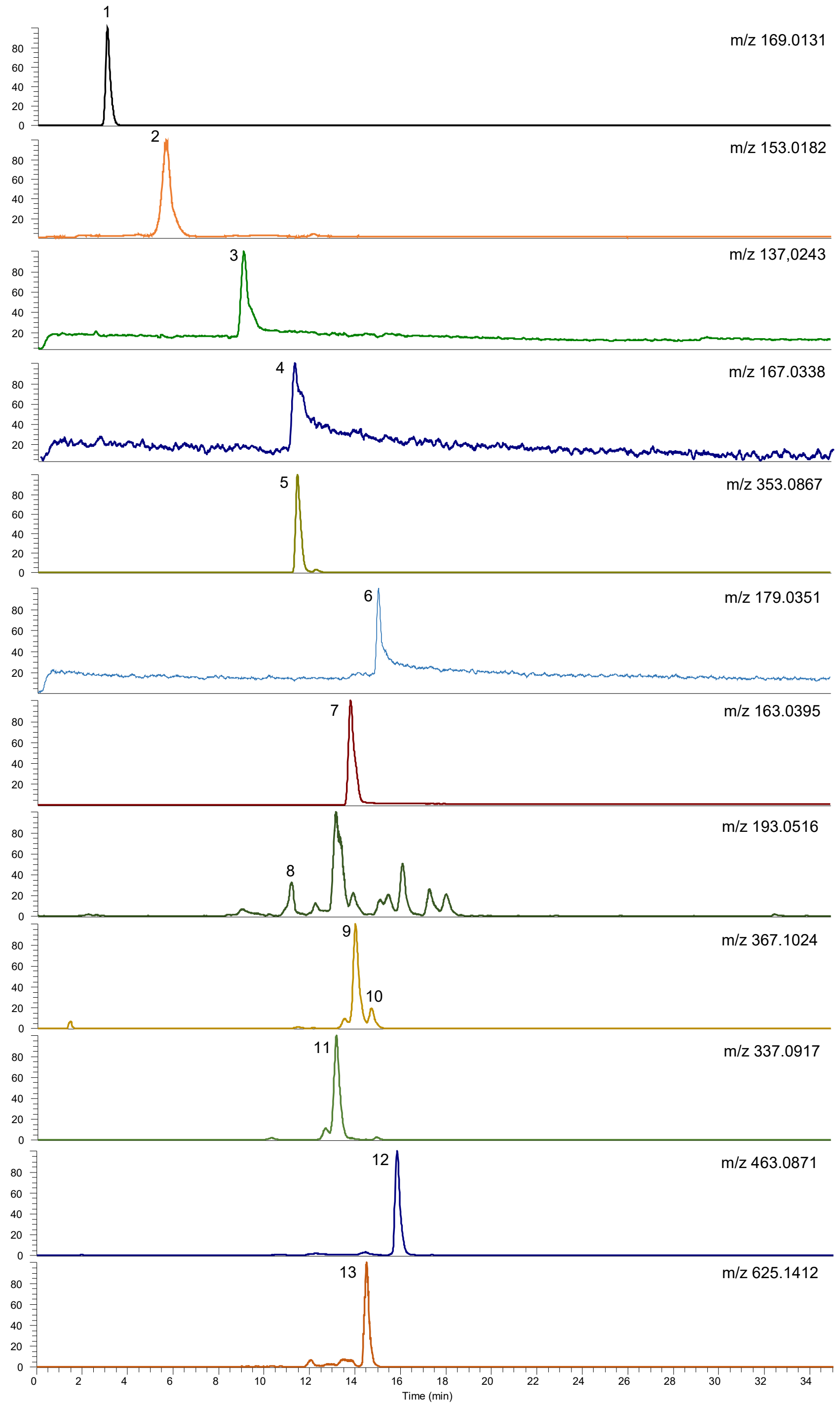

Figure S1. Representative UHPLC-HRMS profile of polyphenols in black carrot. For peak identification see Table S1.

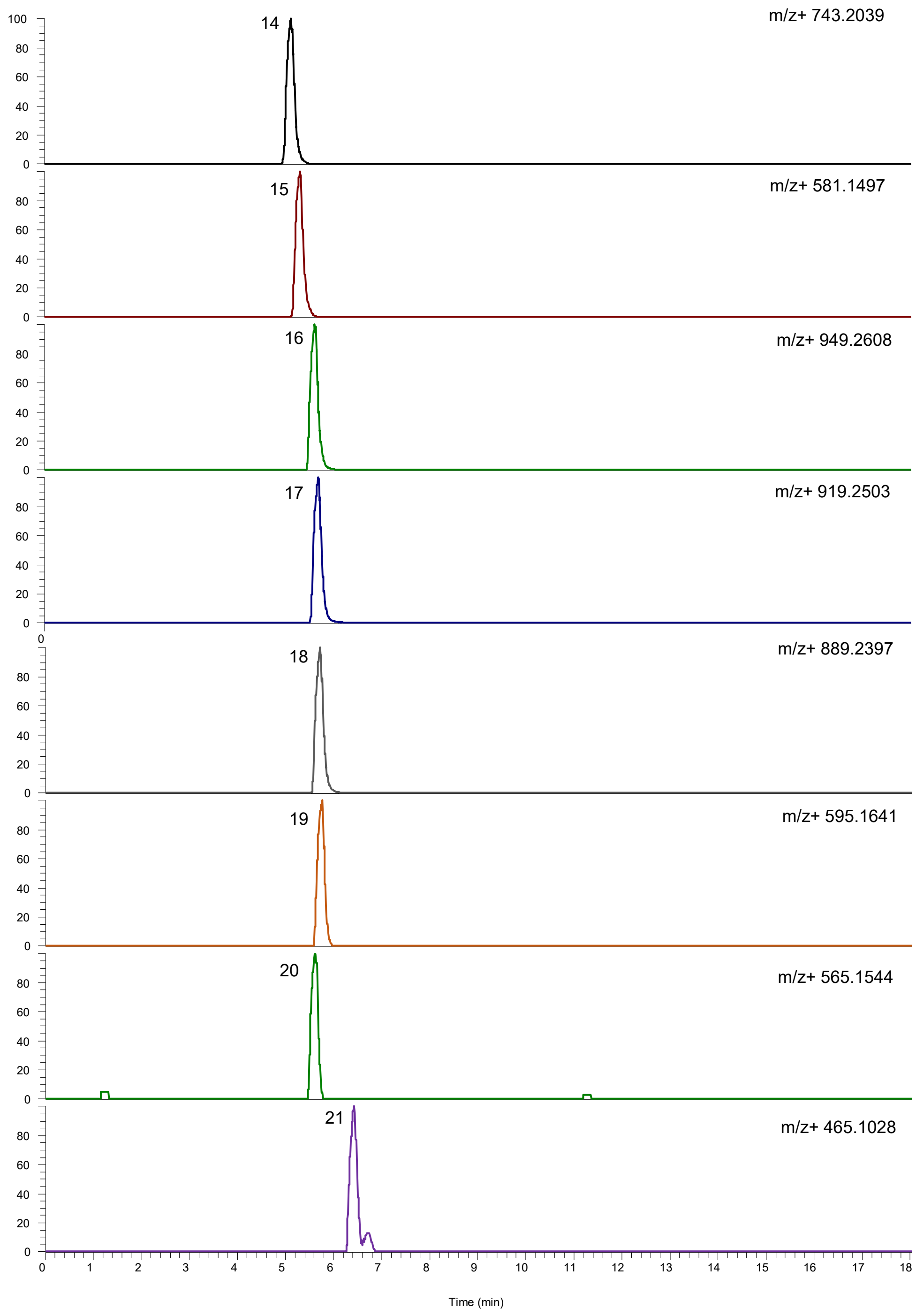

Figure 2. Representative UHPLC-HRMS profile of anthocyanins identified in black carrot. For peak identification see Table S1.
